# Inflammatory index score in children: its relationship with neophobia, dietary quality and anthropometric measurements

**DOI:** 10.1186/s12889-023-17533-3

**Published:** 2024-02-22

**Authors:** Didem Kanısoy, Seray Kabaran

**Affiliations:** Department of Nutrition and Dietetics, Faculty of Health Sciences, Eastern Mediterranean University, T.R. North Cyprus via Mersin 10, Famagusta, Turkey

**Keywords:** Inflammatory index scores, KIDMED scores, Food neophobia, Anthropometric measurements

## Abstract

The aim of this study was to examine the correlation between the Diet Inflammatory Index (DII) scores and dietary quality in children, which was measured by the Mediterranean Diet Quality Index (KIDMED), also neophobia scores and anthropometric measurements. This study was conducted in primary schools in Famagusta, Cyprus. A total of 300 children (150 girls, 150 boys) in the 3rd, 4th and 5th grade were included in the study. The frequency of food consumption was measured to calculate the DII scores. Moreover, neophobia and KIDMED scores were obtained. The KIDMED score is a popular tool that is mostly used as a practical scale to assess adherence to the Mediterranean diet among children. Additionally, anthropometric measurements (body weight, height, waist circumference, neck circumference) were collected. Finally, DII scores were compared with KIDMED scores, neophobia scores, and anthropometric measurements. Anthropometric measurements and body mass index (BMI) values were found to be significantly different (p < 0.05) based on the DII scores. Children with DII scores in the 1st quartile had significantly different anthropometric measurements compared to those who had scores in the 2nd, 3rd, and 4th quartiles (p < 0.05). The DII scores of normal-weight children were higher than those of obese children. A significant negative correlation was observed between KIDMED scores and DII scores of the children (p < 0.05). Furthermore, a significant positive correlation was observed between neophobia scores and DII scores (p < 0.05). Additionally, DII scores were correlated with dietary quality and anthropometric measurements (p < 0.05). The MD enhances the anti-inflammatory properties of the diet; it has clearly demonstrated positive effects on diet quality and anthropometric measurements. Furthermore, the MD is suggested to reduce the risk of chronic diseases as a result of improving DII scores at an early age.

## Introduction

The prevalence of childhood obesity has increased drastically in recent years [1]. It has been reported that more than 340 million children and adolescents aged 5–19 years were overweight or obese in 2016 [2]. High consumption of obesogenic foods is an important risk factor for childhood obesity. Moreover, childhood obesity is an important risk factor for chronic diseases later in life [3, 4].

Dietary inflammation is also known to have a significant impact on the risk of obesity and chronic disease in both adults and children. Studies have shown that a proinflammatory diet in early childhood is associated with a higher BMI z score, waist circumference and adiposity [5–7]. In studies conducted with primary school children, it was seen that consumption of obesogenic foods at meals is high (chips, cola, chocolate, etc.), while consumption of vegetables and fruits is insufficient. Therefore, saturated fat intake increased, and high energy intake and vitamin and mineral deficiencies were observed [8].

It is important to highlight that eating healthy foods with high antioxidant content in childhood can protect against the harmful effects of free radicals on body tissues. As the anti-inflammatory content of a diet increases, the risk of cardiovascular disease and the rate of chronic diseases decrease [9, 10]. For example, the Mediterranean diet (MD) includes vegetables, fruits, legumes, nuts, whole grains, olive oils, fish and moderate wine intake. The MD is mostly used in Mediterranean countries, which have relatively healthy dietary patterns that include relatively high consumption of unprocessed foods and plant foods, such as vegetables, legumes, grains, nuts, fresh and seasonal fruit, breads, and unrefined grains. Recently, the Mediterranean diet has started to gain popularity because it is associated with a healthy lifestyle [11, 12].

The anti-inflammatory or proinflammatory properties of a diet are related to the content of the foods. The MD has been shown to be associated with anti-inflammatory effects, including omega 3 sources and being rich in foods that are a good source of fiber and antioxidants. On the other hand, the Western diet is associated with high saturated fatty acid (SFA) or high carbohydrate intake (mostly obesogenic food consumption) and has shown proinflammatory effects [13, 14].

Furthermore, the variety of nutrients in the MD is high, and it is effective at ensuring adequate growth and development of children and preventing health problems that may occur at later ages. It has been observed that nutritional quality decreases with a Western-style diet. On the other hand, the MD – which is rich in vegetables, fruits, bioactive components (such as flavonoids and polyphenols), fiber and antioxidants – can effectively increase diet quality [11].

The basis of a healthy diet is food variety. Diets with a greater variety of foods are more likely to meet nutritional recommendations than those with a limited variety of foods. Due to the reluctance of children to try new foods, their diet is monotonous and lacks food diversity. Therefore, children have a poor diet quality [15, 16]. Additionally, low inflammatory index scores are associated with high waist circumference and a higher risk of obesity. Factors that negatively affect the inflammatory index, such as high-energy diets and low diet quality, can lead to worse anthropometric outcomes. Additionally, various studies have shown that high BMI values ​​were associated with high energy intake, high fat intake, and low fiber consumption [17, 18].

This study aimed to compare the inflammatory index scores, neophobia score, and KIDMED scores of children and evaluate the relationship between anthropometric measurements and these scores among children aged 8–10 years.

## Method

### Study population and design

This cross-sectional study was conducted between 2019 and 2020 at different primary schools located in the city of Famagusta of the Turkish Republic of Northern Cyprus.

The sample size was calculated based on the following parameters: α = 0.05 and β = 0.20. Thus, the minimum sample size was found to be 285. Schools were visited with the approval of the ethics committee, and 300 students who met the criteria were chosen to voluntarily participate in the study. This study was carried out in accordance with the ethics committee 2019/25 − 03 numbered and 08.11.2019 dated decision. Written informed consent was obtained from all children’s parents or guardians. The present study was also conducted in accordance with the guidelines in the Declaration of Helsinki. Children were selected through a simple random sampling process. The study was conducted by interviewing students face to face using the questionnaire form method. The questionnaire includes 4 sections: the Food Neophobia Scale (FNS), the Mediterranean Diet Quality Index (KIDMED), the 51-item Food Frequency Questionnaire (FFQ) and anthropometric measurements. The dietary inflammatory index was calculated by using the FFQ. The body mass index (BMI) and waist-to-height ratio were calculated by taking anthropometric measurements of body weight, height, waist circumference and neck circumference [19, 20].

## The collection and evaluation of research data

### Anthropometric measurements

#### Body Weight

Body weight was measured to the nearest 0.1 kg by using a standard electronic digital scale with a maximum capacity of 150 kg. Measurements were made with thin clothing, without shoes, and at least 3 h of fasting [21].

#### Height

The distance between the top of the head and the sole of the feet (without shoes) was measured with a tape measure affixed to an apparatus while the child was standing with their back resting on a flat surface, their head upright and their eyes facing straight ahead (Frankfort plane) [21].

#### Waist circumference

While the child was standing up, standing to their right, measurements were taken with a nonstretch tape measure by finding the midpoint between the lowest rib and the cristailiac [22].

#### Neck circumference

Neck circumference (NC) measurements have recently been widely used as a simple scanning method that can be used to determine obesity. NC was measured to the nearest 0.1 cm using a nonelastic tape at the level of the most prominent portion at the thyroid cartilage, with children standing upright and their head held erect, eyes facing forward, and shoulders relaxed [23].

#### Waist-to-height ratio

The waist-to-height ratio was calculated by dividing waist circumference by height. The waist-to-height ratio is an indicator of abdominal fat. It is a more sensitive indicator of health risk than the BMI, and it is easier to measure and calculate than BMI [24].

#### Body mass index (BMI)

The BMI determined by dividing the body weight (kg) by the square of the body height (m^2^). The World Health Organization (WHO) BMI percentile value was calculated according to the children’s age and gender [25].

#### Food Neophobia Scale (FNS)

The Food Neophobia Scale (FNS), which was developed by Pliner in 1992, is the first scale to be developed for measuring neophobia. The FNS consists of 10 item questions about ethnic foods, cultural foods and unfamiliar foods. This scale is extremely reliable (α = 0.88) and is a general neophobia scale that exhibits good validity criteria [21]. The validity and reliability of the Turkish version was confirmed by Elmas in 2019. This version consists of 9 items that are measured using a 5-point Likert scale. The response options are as follows: 1 point indicates strongly disagree, 2 points indicates moderately disagree, 3 points indicates neither agree/nor disagree, 4 points indicates moderately agree and 5 points indicates strongly agree.

By taking the average of the total points obtained after the survey of the population, children scoring above average are classified as neophobic, while children scoring below average are classified as neophilic [26].

#### Mediterranean Diet Quality Index (KIDMED Index)

The KIDMED index was developed by Serra-Majem colleagues. The scale includes 16 questions that evaluate the characteristics of the MD. The KIDMED index includes 12 positive questions and 4 negative questions, where + 1 point is given for the answer yes on positive questions and − 1 point is given to those who answer negative questions as yes. The total scores range from 0 to 12, where scores ≥ 8 points indicate high adherence, scores between 4 and 7 points indicate medium adherence, and scores ≤ 3 points indicate poor adherence to the Mediterranean diet [20].

#### Diet Inflammatory Index (DII)

The 51-item Frequency of Food Consumption was administered to calculate Diet Inflammatory Index scores. Khan et al. designed, developed and validated the Dietary Inflammatory Index for children and calculated the Dietary Inflammatory Index score for 25 nutrients in children [27]. Twenty-five nutrients (vitamin A, thiamine, riboflavin, niacin, B6, folic acid, vitamin B12, vitamin D, vitamin C, vitamin E, β-carotene, energy, carbohydrate, dietary fiber, total fat, saturated fat, monounsaturated fatty acids, polyunsaturated fatty acids, cholesterol, protein, alcohol, iron, magnesium, selenium, and zinc) were evaluated to calculate the inflammatory index of the diet in children. The adult version of this tool assesses more than 45 nutrients. To obtain scores, the standard global average intake is subtracted from the daily consumption of the 25 nutrients and then divided by the world standard deviation intake of this factor (obtained from the data sets of eleven countries). The score is then converted to a value ranging from 0 to 1, and these values are multiplied by 2 and subtracted by 1. Therefore, total scores range from − 1 to + 1. To obtain the DII score specific to a particular food, the DII score for that food is multiplied by the impact score, and all values are then summed. The scores are divided into quartiles [27, 28].

### Statistical analyses

All statistical analyses were performed using Statistical Package for the Social Sciences (SPSS) for Windows version 24.0. P values less than 0.05 were considered to indicate statistical significance. Continuous variables are expressed as the means ± standard deviations (SD), and categorical variables are expressed as numbers (percentages). Variables were tested for normality using the Kolmogorov‒Smirnov test. It was determined that the data set did not follow a normal distribution. Therefore, nonparametric hypothesis tests were used in the study. Comparisons of the basic and anthropometric measurements of the participants were performed. The Mann‒Whitney U test was used to examine the differences between two independent groups, whereas the Kruskal‒Wallis H test was used for comparisons of 3 or more groups. Differences between categorical variables were tested by the chi-square test. Spearman’s correlation analysis was used to examine the relationship between the children’ anthropometric measurements, neophobia, KIDMED and inflammatory index scores.

## Results

Table 1 shows that among the children included in the study, 29.3% were 8 years old, 29.7% were 9 years old, and 41% were 10 years old.

A comparison of the Neophobia scores by gender showed that 46.0% of the female children and 50.67% of the male children participating in the study were neophobic (p > 0.05).

When the KIDMED scores of the female children were analyzed, it was found that 18.0% had poor adherence, 42.67% had medium adherence and 39.33% had high adherence to the diet. On the other hand, among males, 16.0% had poor adherence, 55.33% had medium adherence and 28.67% had high adherence (p > 0.05).

The inflammatory index scores of 26.67% of female students were in the first quartile, 22.67% had scores in the second quartile, 25.33% had scores in the third quartile, and 25.33% had scores in the fourth quartile. The inflammatory index scores of 24.0% of male children were in the first quartile, 26.67% had scores in the second quartile, 24.67% had scores in the third quartile, and 24.67% had scores in the fourth quartile. It was determined that the gender differences were not statistically significant (p > 0.05).

**Table 1:** Demographic data and comparison of the Neophobia, KIDMED and inflammatory index score classifications of children according to gender

	Female		Male		Total		χ^2^	P
	N	%	N	%	N	%		
**Age**								
8 9 10	41 47 62	27,3 31,3 41,3	47 42 61	31,3 28 40,7	88 89 123	29,3 29,7 41		
**Neophobia**								
Neophobic	69	46,00	76	50,67	145	48,33	0,654	0,419
Non-neophobic	81	54,00	74	49,33	155	51,67		
**KIDMED**								
Low	27	18,00	24	16,00	51	17,00		
Medium	64	42,67	83	55,33	147	49,00	5,142	0.076
High	59	39,33	43	28,67	102	34,00		
**Inflammatory** **Index**								
I. Quartile	40	26,67	36	24,00	76	25,33		
II. Quartile	34	22,67	40	26,67	74	24,67	0,724	0,868
III. Quartile	38	25,33	37	24,67	75	25,00		
IV. Quartile	38	25,33	37	24,67	75	25,00		

(Chi square test)

KIDMED: Mediterranean Diet Quality Index

Table 2 shows that there was a statistically significant difference in the inflammatory index scores between different BMI (percentile) classifications (p < 0.05). The inflammatory index scores of children in the extremely thin/underweight groups were higher than the scores of overweight and obese children. In addition, children with a normal weight had higher Inflammatory Index scores than obese children. Finally, no significant difference in the Neophobia scores and KIDMED scores were observed between different BMI (percentile) classifications (p > 0.05).

**Table 2:** Comparison of the neophobia, KIDMED and inflammatory index classifications according to the BMI (percentile) classifications of the children

	BMI (percentile)	N	$\bar x$	S	Min	Max	SO	χ2	P	Difference
**Neophobia Score**	Underweight	14	27,86	7,81	13,00	41,00	128,79	2,530	0,470	
	Normal	140	29,37	6,41	11,00	42,00	146,65			
	Overweight	42	29,40	5,98	16,00	40.00	146,67			
	Obese	104	30,34	6,28	13,00	43,00	160,15			
**KIDMED** **Score**	Underweight	14	5,57	2,50	1,00	9,00	134,93	3,008	0,390	
	Normal	140	6,31	2,36	0.00	11,00	158,45			
	Overweight	42	6,02	2,59	0.00	10.00	153,14			
	Obese	104	5,81	2,35	0.00	10.00	140,83			
**Inflammatory** **Index** **Score**	Underweight	14	2,15	0,79	0,84	3,55	240,21	53,641	< 0.001*	1–3
	Normal	140	1,23	1,23	-2,03	3,58	177,84			1–4
	Overweight	42	0,47	1,16	-1,88	3,23	128,21			2–4
	Obese	104	0,17	1,26	-2,28	2,89	110,62			

*p < 0.05 (Kruskal‒Wallis H test)

KIDMED: Mediterranean Diet Quality IndexBMI: Body Mass Index

Table 3 shows that there were significant differences in body weight, height, waist circumference, neck circumference and BMI values between different Inflammatory Index quartiles (p < 0.05). The body weight, height, waist circumference, neck circumference and BMI values of the children with an inflammatory index classification in the 1st quartile were found to be significantly higher than those of the other children.

**Table 3:** Comparison of anthropometric measurements of students according to inflammatory index classifications

	Inflammatory İndex	N	$\bar x$	S	Min	Max	Average Row	χ^2^	P	Difference
**Weight ** **(kg)**	I. Quartile	76	44,14	10,58	22,20	78,60	217,45	73,229	< 0.001*	1–2
	II. Quartile	74	36,16	8,93	22,30	61,60	156,07			1–3
	III. Quartile	75	32,34	7,74	20,90	53,00	120,33			1–4
	IV. Quartile	75	30,96	7,28	18,50	53,20	107,33			
**Height** ** (cm)**	I. Quartile	76	141,89	8,25	119,00	164,00	205,07	47,241	< 0.001*	1–2
	II. Quartile	74	136,68	8,33	116,00	157,00	153,60			1–3
	III. Quartile	75	133,71	8,58	114,00	153,00	122,53			1–4
	IV. Quartile	75	133,15	8,04	117,00	150.00	120,11			
**Waist** **circumference** **(cm)**	I. Quartile	76	67,84	10,69	32,00	92,00	193,32	31,315	< 0.001*	1–2
	II. Quartile	74	63,58	8,97	49,00	96,00	155,57			1–3
	III. Quartile	75	61,67	8,51	49,00	87,00	132,71			1–4
	IV. Quartile	75	59,75	6,86	47,00	84,00	119,90			
**Waist/Height** **ratio**	I. Quartile	76	0,48	0.07	0,27	0,67	170,21	6,962	0.073	
	II. Quartile	74	0,47	0.06	0,34	0,70	153,75			
	III. Quartile	75	0,46	0.06	0,37	0,64	142,33			
	IV. Quartile	75	0,46	0.07	0,37	0,90	135,49			
**Neck** **Circumference**	I. Quartile	76	31,99	11,43	26,00	128,00	198,79	45,998	< 0.001*	1–2
	II. Quartile	74	29,68	2,51	25,00	37,00	160,74			1–3
	III. Quartile	75	28,81	2,53	23,00	36,00	133,83			1–4
	IV. Quartile	75	28,09	2,07	24,00	33,00	108,13			
**BMI** **(kg/m** ^**2**^ **)**	I. Quartile	76	21,77	4,09	15,20	32,80	209,19	59,065	< 0.001*	1–2
	II. Quartile	74	19,17	3,52	14,30	32,40	156,78			1–3
	III. Quartile	75	17,93	2,91	13,50	25,00	128,60			1–4
	IV. Quartile	75	17,21	2,88	12,80	28,10	106,73			

*p < 0.05 (Kruskal‒Wallis H test)

BMI: Body Mass Index

Table 4 shows the significant negative correlations between the inflammatory index scores of the included children and their body weight, height, waist circumference, waist/height ratio, neck circumference and BMI values ​​(p < 0.05). There was also a significant positive correlation between children’ Neophobia scores and Inflammatory Index scores (p < 0.05).

A significant negative correlation was observed between children’ Neophobia scores and KIDMED scores (p < 0.05). There was a significant negative correlation between the KIDMED and the inflammatory index scores (p < 0.05). On the other hand, there was no statistically significant correlation between children’ Neophobia and KIDMED scores and their anthropometric measurements (p > 0.05).

**Table 4:** Correlations between children’ anthropometric measurements and neophobia, KIDMED and inflammatory index scores as well as correlations between the scores

		Neophobia Score	KIDMED Score	Inflammatory Index Score
**Weight (kg)**	R	0,038	-0,113	-0,488
	P	0,515	0,050	< 0.001*
**Height (cm)**	R	-0,032	-0,055	-0,370
	P	0,581	0,343	< 0.001*
**Waist Circumference (cm)**	R	-0,018	-0,052	-0,339
	P	0,762	0,371	< 0.001*
**Waist/Height** **Ratio**	R	0,030	-0,057	-0,174
	P	0,601	0,325	< 0.001*
**Neck** **Circumference**	R	-0,011	-0,015	-0,404
	P	0,852	0,800	< 0.001*
**BMI** **(kg/m** ^**2**^ **)**	R	0,053	-0,101	-0,456
	P	0,359	0,080	< 0.001*
**Inflammatory Index Score**	R	0,147	-0,182	1
	P	< 0.001*	< 0.001*	
**Neophobia Score**	R	1	-0,490	
	P		< 0.001*	
**KIDMED Score**	R		1	
	P			.

*p < 0.05 (Spearman correlation analysis)

^*+*^ *p < 0.05 (Kruskal‒Wallis H test)*

KIDMED: Mediterranean Diet Quality Index

BMI: Body Mass Index

## Discussion

The inflammatory index among in children positively affects diet quality and anthropometric measurements [17, 18, 29, 30]. Our findings showed that there was a significant negative correlation between the KIDMED scores of the children and the inflammatory index scores. The most significant result of the study is the determination of a significant relationship between children’s anthropometric measurements and the Inflammatory Index. Currently, inflammation poses a threat to future health because it is related to many diseases. Therefore, it is important to improve dietary patterns in childhood.

Currently, the MD has been proposed as a valuable nutritional intervention. The MD is characterized by high consumption of vegetables, fruits, nuts, whole grains and olive oil; moderate consumption of fish, poultry and red wine; and limited intake of sweets, red meat and dairy products [31]. Previous research has suggested the beneficial role of the MD in improving glycemic control, controlling blood pressure and protecting against coronary heart diseases [32, 33]. Moreover, the MD positively modulates the immune system and inflammatory mediators, thereby affecting health. However, the Western-style diet, which is widespread among adolescents, is characterized by unhealthy dietary patterns, such as high contents of red meat, processed products, saturated fat, alcohol and sugar. This dietary pattern leads increases oxidative stress and facilitates pro-inflammatory effects. Current research shows that diets with high antioxidant content can play an important role in modulating inflammation [34].

In our study, children showed a higher level of dietary adherence: adherence was poor in 17% of students, moderate in 49%, and high in 34%. Korkmaz et al. examined 900 children in Ordu Province and found results that were similar to ours. They found that 45.6% of children showed moderate adherence, only 18.7% had high dietary adherence, and 35.7% had poor adherence to the MD [35]. In a study conducted in Northern Italy, primary school children’s adherence to the MD was generally similar to our study. Most of the children had moderate adherence to the MD. Nonetheless, 20.7% of children had poor adherence, 60.3% had moderate adherence, and 19% of the students had high adherence to the MD [36]. In the current prospective cohort study in Spanish children, 40.4% had high adherence, 50% had moderate adherence, and 9.6% had poor adherence to the MD [37].

The MD is popular because it is one of the diets that positively affects health. Additionally, the MD is associated with the weight status of both children and adolescents [38, 39]. High adherence to the MD has been observed to reduce the likelihood of being overweight or obese in both boys and girls by 30%. Previous studies have highlighted the importance of dietary patterns in the prevention of obesity among adolescents [40]. Although, in our study, children with medium and high adherence to the MD were underweight and normal weight (Table 2), we found that there was a nonsignificant negative correlation between KIDMED scores based on the BMI (percentile) classification of the included children(p > 0.05).

Obese children and adolescents are more likely to be highly obese in adulthood [3]. According to data from the WHO, the prevalence of overweight and obesity among children and adolescents aged 5–19 was 4% in 1975 and exceeded 18% in 2016. In our study, 46.66% of the children had a normal weight, 14% were overweight and 34.66% were obese (Table 2). A large-sample longitudinal study in Spain showed that obesity dramatically increased in the years between 2005 and 2017; 64% of children had a normal weight, 21.4% were overweight, and 14.2% were obese [41]. Previous research has shown that children who are obese between the ages of 7 and 11 are 5 times more likely to be obese in adulthood than children who are not obese during that period [42]. A previous study evaluated the effects of pediatric obesity and its complications on life expectancy [42, 43]. Regarding the association between obesity and dietary patterns, a Western-style dietary pattern is positively associated with adiposity and inflammation in African-American adolescents [44]. Children with overweight or obesity are hyperinsulinemic and have approximately 40% less insulin-stimulated glucose than children with a normal weight. The development of T2DM is closely related to insulin resistance. The relationship between obesity and inflammation starts early in life and causes advanced chronic diseases [45].

Dietary patterns with low anti-inflammatory content are a major risk factor for childhood obesity. Previous studies have shown that obesity-related adipose tissue dysfunction develops in early childhood and is associated with proinflammatory biomarkers. Obese adolescents have been observed to have higher inflammatory markers than adolescents with a normal weight [46]. Recent studies have shown that high BMI values ​​were associated with a decrease in inflammatory index scores. This finding supports the hypothesis of an association between low-grade systemic inflammation early in life and subsequent obesity [47, 48]. Our findings revealed a statistically significant difference between the inflammatory index scores based on the BMI (percentile) classification of the children (p < 0.05). Children with a normal weight have higher Inflammatory Index scores than obese children (Table 3). Previous studies have shown that the inflammatory index score is higher in obese and slightly obese patients [17, 18, 29, 30]. A decrease in healthy eating patterns has been shown to be associated with increased obesity. Additionally, high consumption of fast-food foods has been shown to be associated with a higher risk of obesity [17]. In summary, obesity is associated with systemic low-grade inflammation in both children and adults, and an increase in the anti-inflammatory content of the diet is associated with a reduced risk of cardiovascular disease as well as a reduced incidence of chronic diseases [9, 10, 49]. Therefore, in order to support a healthy weight, children and adolescents should be advised to consume anti-inflammatory foods/nutrients (such as fruits and vegetables); to consume foods containing omega 3 fatty acids and foods rich in fiber, flavonoids, zinc, magnesium and selenium; and to avoid or limit the consumption of processed foods that are rich in saturated fatty acids (SFA), trans-fatty acids, and cholesterol [18].

In our study, it was determined that the difference between body weight, height, waist circumference, neck circumference and BMI values of the included children was statistically significant according to the Inflammatory Index classification (p < 0.05) (Table 2). Several studies have shown that an anti-inflammatory diet is an important aspect of the effective prevention and treatment of obesity [50, 51]. The findings of the PREDIMED study showed an association between the anti-inflammatory effects of the DII, the intake of a healthy diet and a higher adherence to the MD [52]. As expected, we observed that the DII score was inversely associated with the intake of a healthy dietary pattern and adherence to the MD. Moreover, the anti-inflammatory effect of the diet increased as adherence to the MD increased (Table 4).

It was expected that there would be a difference in food neophobia depending on the decrease in dietary diversity, but such a difference was not observed in our study (Table 4). However, a difference in neophobia scores was observed between male and female children. Similar results were reported by de Almeida et al., who found that boys were significantly more neophobic than girls [53]. Factors such as ethnicity and local dietary patterns could explain these differences in neophobia.

This study has some limitations that should be used to guide future studies, including the small sample size and the lack of a 3-day food consumption record. For future studies, randomized controlled trials that examine dietary inflammatory indices in obese and overweight children will provide meaningful results. In this manner, by increasing the consumption of anti-inflammatory foods, the inflammatory diet index score can be determined for weight loss and prevention of obesity.

## Conclusion

The results confirm the hypothesis that healthy dietary patterns, such as the MD, are significantly related to dietary anti-inflammatory effects in children. In addition, it was observed that there was a relationship between dietary quality and anthropometric measurements with the DII. In summary, a healthy dietary pattern is important for improving health consequences in early childhood. Overall, higher dietary variety and higher dietary quality are associated with increased anti-inflammatory contents in children diet. The widespread use of inflammatory index scores by dietitians to evaluate obesity and diet is a positive development for increasing the anti-inflammatory effects of diets. A better quality of life can be achieved by improving diet quality practices and evaluations. To explore the relationship between neophobia and DII scores more clearly, longer follow-ups with a higher number of participants may provide more meaningful results.

## Data Availability

The data that support the findings of this study are available from the authors upon reasonable request and with the permission of Didem Kanısoy. Restrictions apply to the availability of these data, which were used under license for the current study and are thus not publicly available.

## Abbreviations

KIDMED: Mediterranean Diet Quality Index
DII: Diet Inflammatory Index Score
BMI: Body Mass Index
MD: Mediterranean Diet
FNS: Food Neophobia Scale
NC: Neck circumference
FFQ: Food Frequency Questionnaire
